# Integrative Genetic Analysis of Alcohol Dependence Using the Genenetwork Web Resources

**Published:** 2008

**Authors:** Robert W. Williams, Lu Lu

**Keywords:** Alcoholism, genetic theory of alcohol and other drug use, alcohol-related phenotypes, genetic factors, environmental factors, genetic traits, DNA variants, candidate genes, genetic strains, animal models, laboratory rats, laboratory mice, GeneNetwork

Not long ago, the idea that alcoholism had a genetic basis—even in part—was highly controversial (Goodwin 1971, 1975). Over the past 35 years, however, the contribution of genetic factors has become conventional wisdom. This change to a large extent results from compelling experimental studies utilizing inbred and recombinant inbred (RI) strains of rats and mice (e.g., Crabbe et al. 1983; Crabbe 1983; McClearn 1972). Over the last decade, the focus of genetic research in the alcohol field has shifted away from epidemiological and statistical estimates of alcoholism heritability to molecular studies of single gene variants that have explanatory, predictive, and even therapeutic utility. Large-scale genetic studies such as the Collaborative Study on the Genetics of Alcoholism (COGA) have highlighted variations (i.e., polymorphisms) in numerous genes that are related to alcohol use, abuse, and risk of dependence (for a review, see Edenberg and Foroud 2006). And the list of candidate genes keeps growing. However, just compiling lists of such genes is not satisfying. What is needed is an in-depth understanding of how DNA variants cause differences in alcohol dependence, especially in the context of a myriad of environmental contributing factors, such as diet, stressors, and previous drinking history. This article summarizes some of the challenges associated with generating this understanding and presents a Web-based resource that may aid researchers in better understanding the mechanisms through which alcohol acts on the body and how different genes influence this process.

## Challenges Associated With Studying the Relationship Between Genes and Alcohol Response

With an ever-expanding list of candidate genes at their hands, alcohol researchers now face the challenge of systematically and efficiently dissecting the mechanisms by which differences in the DNA sequence can modulate responses to alcohol. This is particularly difficult because sequence variations usually only have small effects, with changes in any one gene typically accounting for less than 5 percent of the variation in the characteristic or behavior (i.e., the phenotype) studied. Furthermore, genes and their products are parts of complex biological networks that can resist conventional reductionist analyses.

To identify the genes and genetic variations that contribute to alcoholism development, researchers must rely on experimental approaches, often using rodent models that allow them to reduce confounding environmental effects. Many of these models involve inbred rodents, which essentially are groups of genetically identical animals that can be maintained over many generations. Therefore, it is possible to resample a particular inbred line many times, under the same or different environmental conditions (e.g., under high or low stress levels). These and other models enable investigators to validate associations between certain DNA sequences and specific responses to alcohol and, more importantly, to test for causality among these relations. To this end, a suite of techniques allowing analyses at diverse levels (e.g., genomic, cellular, pharmacological, neurophysiological, behavioral, and bioinformatics levels) now can be exploited to analyze complex networks that link differences in DNA sequence to such phenotypes as risk of dependence and relapse.

Despite this progress, two important problems remain. First, the results obtained with animal models will not necessarily translate well to human populations with their mixed genetic backgrounds. This problem may be solved by studying populations of rodent strains whose level of genetic complexity mimics that of human populations. Using this “population” model approach, results will not hinge on animals with a single genetic makeup (i.e., genotype) or individual strains but will reflect a consensus with validity across many strains. The second issue is how to go about integrating the many different types and levels of data that can be acquired from animal models. The questions to be answered often require a depth and breadth of data types that are well beyond the reach of an individual laboratory. An initial step in solving this problem is to develop resources, tools, and collaborations that enable many investigators to share not only their research findings but also their underlying data. This is one of the main goals of the Integrated Neuroscience Initiative on Alcoholism (INIA) program, which is sponsored by the National Institute on Alcohol Abuse and Alcoholism (NIAAA).

## The GeneNetwork Web Resource

Another approach to helping researchers integrate data obtained at different levels and in different organisms is GeneNetwork,^1^ a Web site and resource (www.genenetwork.org) that provides access to a wide variety of data (e.g., genotypes, phenotypes, messenger RNA [mRNA] expression levels) from populations of different strains of mice and rats. The available rodent strains are grouped together into families that share common origins. For example, the BXD family consists of a set of 80 recombinant inbred (RI) strains that all can be traced back to matings between two inbred mouse strains called C57BL/6J and DBA/2J. The equally large LXS family of strains traces back to a cross between two strains of mice that differ in their sensitivity to alcohol-induced sedation (i.e., inbred long sleep [ILS] and inbred short sleep [ISS] strains). Both of these families of strains are ideal resources for studying the role of genetic factors on effects of alcohol because of the distinct differences between the parental strains. Thus, C57BL/6J mice typically are avid drinkers, whereas DBA/2J mice require coercion to ingest alcohol. Similarly, ILS and ISS animals differ markedly in the length of time they need to recover from an acute alcohol injection.

**Figure 18:**
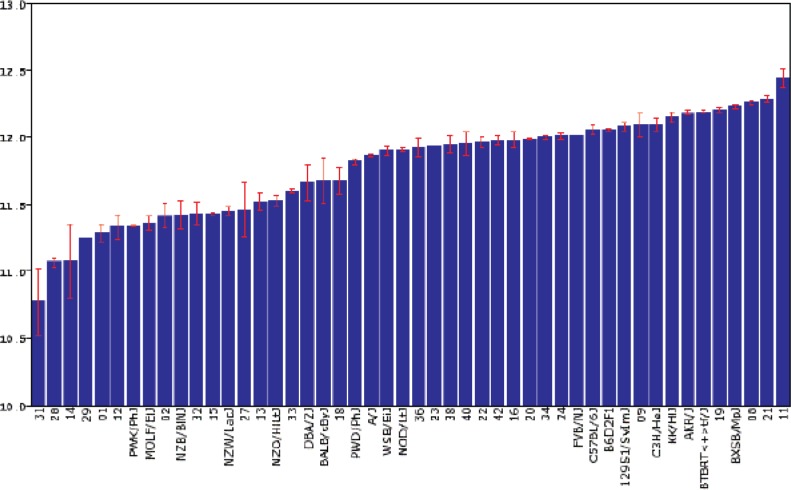
Expression of the *Gabra1* gene, which encodes a component of the GABA_A_ receptor for the neurotransmitter γ-aminobutyric acid (GABA), in a brain region called the striatum of a set of 48 inbred strains of mice. These included 31 strains derived from the BXD family of recombinant inbred strains, which were initially generated by mating C57BL/6J and DBA/2J mice. Each strain is represented by a separate bar. One unit change in the y-axis represents a two-fold difference in the steady-state levels of the *Gabra1* mRNA in the striatum. Strains that had inherited the *Gabra1* gene from the C57BL/6J parents generally exhibited higher expression of the gene than other strains.

The parental strains and their progeny RI strains of the BXD and LXS families have been well characterized at the genetic level. For example, it now is a trivial task to find all known sequence differences between the C57BL/6J and DBA/2J strains in a mouse gene called *Gabra1*, which encodes a component of a receptor for the brain signaling molecule (i.e., neurotransmitter) γ-aminobutyric acid (GABA).^2^ The idea that *Gabra1* expression would be related to alcohol phenotypes is based on a large body of pharmacological data (e.g., Enoch et al. 2008). In this gene, researchers have identified 123 small changes in the DNA (i.e., single nucleotide polymorphisms [SNPs]) between the two strains, 9 of which are located in regions of the gene that actually serve as templates for the GABRA1 protein (i.e., in exons). The GeneNetwork resource allows researchers to investigate several questions regarding the relevance of these genetic differences between the C57BL/6J and DBA/2J strains. For example, do these differences in *Gabra1* sequence affect the gene’s expression in the brain? The answer is “yes” because expression data from two brain regions (i.e., the striatum and the hippocampus) indicate that expression is higher in those BXD strains (roughly 50 percent) that have inherited their *Gabra1* gene from the C57BL/6J parent (figure 18).

**Figure 19:**
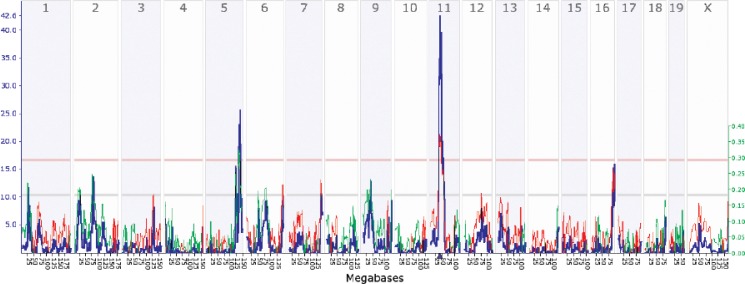
A genetic map of *Gabra1* expression in a brain region called the hippocampus of mice from the BXD family of recombinant inbred strains. The x-axis lists mouse chromosomes 1 through X. The y-axis provides a measure of linkage (LRS = likelihood ratio statistic) between variation in *Gabra1* expression and each part of the mouse genome. A prominent peak on chromosome 11 provides strong confirmation that sequence differences near the *Gabra1* gene (which is located on that chromosome) control *Gabra1* expression. Secondary peaks on chromosomes 5 and 16 suggest that DNA sequences in those regions likely also modulate expression of the *Gabra1* mRNA.

Similarly, the tools available on the GeneNetwork Web site allow investigators to verify that the observed differences in expression actually are caused by sequence variants in or near the *Gabra1* gene by determining which area of the mouse chromosomes is associated with variation of mRNA expression (see figure 19). These analyses found that the greatest association between a chromosomal region and *Gabra1* mRNA expression existed for a region on chromosome 11 that corresponds precisely to the location of *Gabra1*; additional findings confirmed the validity of this result.

## Future Outlook

The analyses described above have demonstrated the potential of resources such as the GeneNetwork Web site in identifying specific genes that may contribute to alcohol-related phenotypes. The next step then is to determine in more detail how differences in the sequence and expression of these genes lead to differences in alcohol-related phenotypes. To help address these questions, GeneNetwork contains information on hundreds of alcohol-related phenotypes, primarily for the BXD RI panel; these data were freely contributed by numerous investigators (as were gene expression data). For example, analyses using the database found that the alcohol-related phenotype that showed the strongest correlation with *Gabra1* expression was alcohol acceptance (Crabbe et al. 1983). This correlation subsequently was confirmed using an RI dataset. Detecting these sorts of associations between certain phenotypes and the expression of individual genes, gene clusters, and gene networks all can be accomplished with the tools available at GeneNetwork.

## Footnotes

1 GeneNetwork is sponsored by different grants, including grants from INIA and a Human Brain Project funded jointly by NIAAA, the National Institute on Drug Abuse, and the National Institute of Mental Health.

2 GABA is the major inhibitory neurotransmitter in the mammalian brain. It acts mainly at a receptor called GABA_A_ receptors. Each GABA_A_ receptor complex is composed of five subunits. At least 16 distinct subunits of GABA_A_ receptors have been identified, including GABRA1.
